# Optimal Environmental Siting of Future Wind Turbines in the North Sea

**DOI:** 10.1021/acs.est.4c03861

**Published:** 2024-12-19

**Authors:** Chen Li, Bernhard Steubing, Joeri Morpurgo, Arnold Tukker, José M. Mogollón

**Affiliations:** † Institute of Environmental Sciences (CML), Leiden University, P.O. Box 9518, Leiden 2300 RA, The Netherlands; ‡ Netherlands Organization for Applied Scientific Research, P.O. Box 96800, Den Haag 2509 JE, The Netherlands

**Keywords:** offshore wind energy, the North Sea, material use, life cycle environmental impacts, biodiversity, spatial planning, optimization, technological development

## Abstract

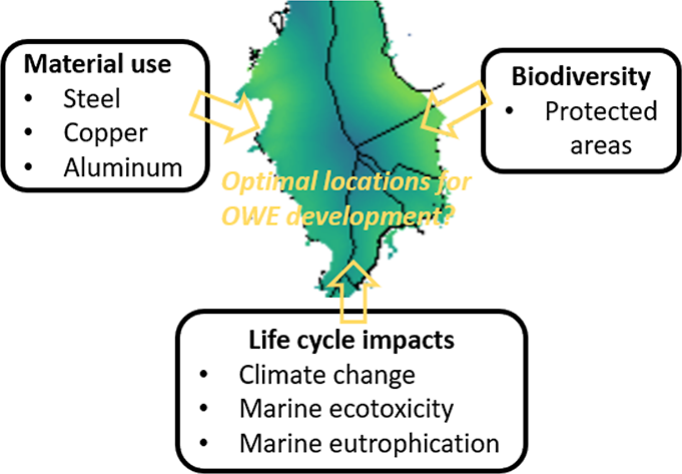

Offshore wind energy (OWE) represents a key technology for achieving a sustainable energy transition. However, offshore wind farms (OWFs) can impact the environment via installation, operation, maintenance, and decommissioning activities together with the raw materials and energy required for their manufacturing. This study assesses the material and carbon footprint of potential OWF locations in the North Sea for various possible future technology developments. We find that better sitings could save up to ∼0.11 kg (∼65%) of steel, ∼ 0.16 g (∼31%) of copper, and ∼6.44 kg (∼26%) of embodied CO_2_-eq per MWh of electricity produced compared to the status quo setups. Nearshore regions of the North Sea, particularly the eastern and northwestern areas, have the lowest CO_2_-eq per MWh of electricity produced due to favorable wind resources. Developing an OWF in the central North Sea requires more copper and aluminum due to large distances to shore and thus incurs higher embodied CO_2_-eq per MWh. These areas also overlap with several protected areas and thus remain the least favorable for OWE development. The future emergent OWE technological developments for 2040 such as the installation of larger turbines with an extended lifetime alone could, on average, lead to reductions of ∼0.06 kg in steel demand (∼35%), ∼ 0.15 g in copper demand (∼31%), and ∼10.97 kg of CO_2_-eq (∼41%) per MWh produced. Future OWFs incorporating these technological developments, when placed in the most suitable locations, have the potential to substantially lower OWF environmental impacts across the full turbine life cycle.

## Abstract 2

Our study represents a pioneering effort in quantifying site-specific benefits and drawbacks concerning material requirements and climate impacts for the current and future wind energy development in the North Sea.

## 1 Introduction

**Figure 1:**
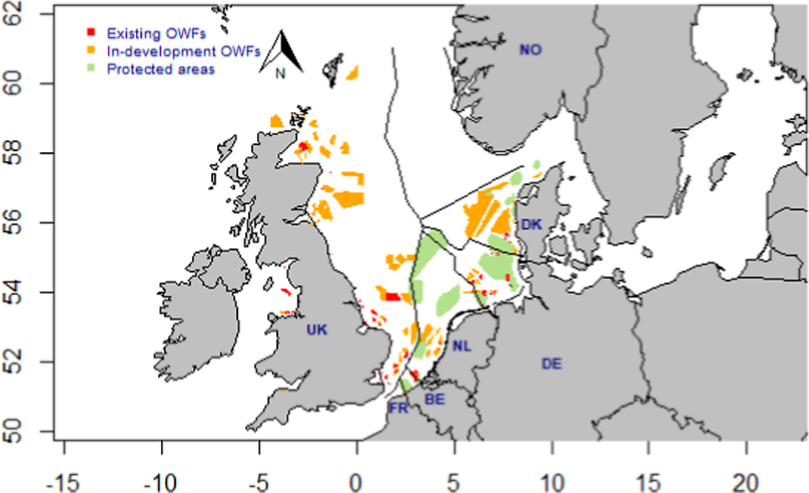
Overview of the North Sea, including the Norwegian (NO), Danish (DK), German (DE), Dutch (NL), Belgian (BE), French (FR), and United Kingdom (UK) exclusive economic zones (EEZs). The map includes the existing (red) and in-development (including under construction, approved, and planned, orange) offshore wind farms (OWFs) and protected areas (green).

Offshore wind energy (OWE) is increasingly being implemented in many coastal regions.^1^ There is a growing need to accurately pinpoint and understand the environmental footprint related to OWE deployment.^2^ Particularly, the location of offshore wind farms (OWFs) may create trade-offs throughout their life cycle between electricity production and material, carbon, and biodiversity footprints. The North Sea is an attractive sea basin for OWE development due to strong and continuous winds, relatively shallow water,^3^ and proximity to extensive energy and electricity markets.^4^ Consequently, the North Sea is globally at the forefront of OWE development. Between 2011 and 2020, the overall installed capacity in the North Sea tripled to ∼19 GW, reaching two-thirds of the global installed OWE capacity.^5^ In a continuous push to harvest OWE, plans for the North Sea region now aim at 175 GW of installed capacity by 2040,^5^ which is roughly a quarter of the contemporary European Union annual electricity demand (∼2800 TWh).^6^ These large-scale OWFs will cover roughly one-fourth of the total surface area (187,500 km^2^) of the North Sea (Figure 1). They will also need substantial quantities of bulk and critical raw materials for the manufacture of turbines, foundations, and transmission components. Furthermore, the manufacturing, installation, operation, maintenance, and decommissioning of OWFs have additional direct and indirect impacts on the environment via energy use in material manufacturing, seabed occupation, and material and personnel transport.

To ensure the most environmentally friendly OWE deployment, an optimization of various factors and trends needs to be taken into account: (1) Spatial location: enhanced wind resources are often encountered in the northern parts of the North Sea and farther offshore. Water depths also vary, with shallower regions in the south gradually deepening toward the northern areas. Several protected areas are scattered throughout the North Sea. (2) OWE technology development: greater turbine capacity depends on enlarging the turbine size, requiring an increase in rotor diameter, hub height, and, consequently, the larger size of support (e.g., foundation) structures.^7^ Further, rapidly advancing technologies change the material composition and type of components (e.g., generator, rotor, and blades)^7,8^ and associated material use and energy use throughout the whole supply chain. Lastly, turbine lifetimes increase with time.

Here, we analyze the spatial/geographical siting choices and the technological improvements of OWFs in the North Sea to assess the environmental footprint per MWh of electricity produced across the full turbine life cycle. We include end-of-life, material demand, impacts on global warming, and potential impacts on biodiversity. We analyze this footprint in specific locations by considering multiple geographical factors, including wind speed, water depth, and distance from shore. We compare present-day technologies (2020) and estimates of a future emergent technology mix (2040), characterized by, e.g., enlarged turbine sizes, longer lifetimes, and improved component technologies. We estimate material demand, including steel, copper, and aluminum required for the manufacturing of the nacelle, rotor (including blades), and tower using a dynamic material flow analysis.^7^ We calculate the material use for the foundation and transmission infrastructure by considering the water depth and distance from shore, respectively. Using the site-specific material demand per MWh of electricity production, we calculate the life cycle environmental impacts by using a prospective life cycle assessment model as described in earlier work.^8^ Our model includes life cycle impacts for climate change (in this paper expressed in GWP100 CO_2_-eq), marine ecotoxicity, and marine eutrophication (expressed as METPinf kg 1,4-DC-eq and MEP kg *N*-eq, respectively^8^). We also investigated the potential impacts on biodiversity by screening overlap with protected areas of potential OWE locations. Increasing overlap with protected areas suggests potentially higher impacts on biodiversity, while fewer overlaps indicate potentially lower impacts. This comprehensive analysis can be used for the strategic planning of OWE locations, providing the major hotspots in environmental impacts for the North Sea and thus the least impactful locations for OWFs per MWh of electricity produced.

## 2 Materials and Methods

We use the marine region map^9^ as the base map for the North Sea in our geographical information system (GIS)-based analysis.^10^ We add the exclusive economic zone (EEZ) boundaries, existing and in-development (including under construction, approved, and planned) OWFs, and protected areas^11^ to the base map (Figure 1). The environmental footprints per MWh of electricity produced by the OWE, including material demand (steel, copper, and aluminum) and environmental impacts (climate change, marine ecotoxicity, and marine eutrophication) throughout the full turbine life cycle, are calculated as the ratio of the footprint and electricity output (EO) across a turbine’s full life cycle and normalized to one MWh. We calculate the average footprint for each existing and in-development OWF by EEZ by considering geographical factors, including wind speed, water depth, and distance from shore, based on the current (2020) technology mix. We also calculated the average footprint for each EEZ by comparing the current and future (2040) emergent OWE technology mix. We calculate optimal siting maps based on the 25% (occupation areas of 175 GW capacities) lowest values of steel and copper demand and climate change, respectively. All analyses are performed using the R Statistical Software v4.2.0.^12^

### 2.1 Calculation of Electricity Output

The electricity output (EO) of a single wind turbine throughout its full lifetime is calculated based on the turbine’s nominal capacity (NC), a simplified Rayleigh statistics,^13^ and lifetime (LT) (see eq 1). The Rayleigh statistics is a function of rated wind speed (R) and mean annual site-specific wind speed (WS) at hub height (shown in eq 1; see details in 2.5 and 2.1 of the Supporting Information).
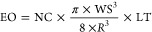
1

We use dynamic parameters for nominal capacity (∼6.5 MW in 2020 and ∼15.6 MW in 2040) and lifetime (20 years in 2020 and 25 years in 2040), with a summary provided in Table 1. We use the rated wind speed of approximately 10.59 m/s from IEA’s 15 MW reference offshore wind turbine.^14^ We normalize the footprints to one MWh by dividing the total electricity output across a turbine’s lifetime.

**Table 1:** Overview of the Current (2020) and Future (2040) Emergent Technology Mix

technological factor	parameter	current (2020) technology mix	future (2040) emergent technology mix
turbine size^7,8^	nominal capacity (MW)	∼6.5	∼15.6
	rotor diameter (m)	148	262
	hub height (m)	115	156
turbine lifetime (yrs)^7^		20	25
electricity output per turbine (GWh)	electricity output across the full turbine lifetime, based on the average wind speed in the North Sea	∼559	∼1251
component technology^8^	nacelle	permanent magnet (PM) free generator technologies still dominate the market	market share of PM-based generator technologies is rising
	blades	both glass and carbon fibers are used	more carbon fibers will be used
	tower	only steel towers are used	hybrid towers will be used
maintenance times^8^	unscheduled and scheduled maintenance	two times unscheduled and four times scheduled maintenance	two times unscheduled and two times scheduled maintenance
replacement rates^8^	annual replacement rate	high annual replacement rates (∼5%)	moderate annual replacement rates (∼2.5%)
transportation means^8^		no additional helicopters required	20% of wind turbines were assumed to be supported by helicopters
background system change^8^		less green energy mix	greener energy mix

### 2.2 Calculation of Material Demand

We evaluated one bulk material (steel) and two major metals (copper and aluminum) that are incorporated in different components of the wind turbines. The OWE foundation structures are primarily made of steel, with their specifications determined by the water depth. We calculate the steel demand for the foundations by considering site-specific water depth (see details in 2.2 in Supporting Information). Copper and aluminum are contained in transmission infrastructures, mainly in cables. We calculate the material requirement for transmission infrastructure by considering distance from shore^15^ (see details in 2.2 in Supporting Information). We add material demand results for the nacelle, rotor (including blades), and tower from a dynamic material flow analysis.^7^

### 2.3 Calculation of Life Cycle Impacts on the Climate

We calculate three life cycle impact categories: climate change, marine ecotoxicity, and marine eutrophication (see 2.3 in Supporting Information for the justification) using the ReCiPe Midpoint (H) V1.13^16^ approach in a prospective life cycle assessment model.^8^ We include an advanced technology foreground scenario and an SSP2-RCP2.6 background scenario^17^ (see details in 2.6). We use dynamic parameterized LCIs that include detailed, full supply chains for state-of-the-art and perspective technologies for four OWE components: the nacelle, rotor, tower, and foundation. These LCI processes include the bulk and key materials requirements (23 chemical elements in total), as well as the energy consumption in the manufacturing of OWE components, vessel operations during OWE component assembly, construction of final units, cable laying, operation and maintenance (O&M), and decommissioning.^8^ These LCI processes were further categorized into water depth-dependent, distance from shore-dependent, and additional processes. Water depth-dependent processes, namely, foundation-relevant processes, are converted to impacts per meter and adapted to water depth. Distance from shore-dependent processes, namely those related to export cables, are updated by multiplying material demand per km with the distance from shore. Processes related to installation, O&M, and decommissioning are converted to impacts per km and adapted to the distance from shore (see details in 2.6 and source data).

### 2.4 Estimation of Impacts on Marine Biodiversity

We assess OWE impacts on marine biodiversity by screening the overlaps of potential OWE installations with the Natura 2000 protected areas.^18^ We assume that increased spatial overlap with protected areas poses a greater risk of biodiversity loss. We discuss the impacts of the OWE-related biodiversity in 4.1.

### 2.5 Modeling of Geographic Factors (Spatial Perspective)

For each grid, we use the mean annual wind speed (m/s) from NEWA^19^ at 200 m as this height is close to the wind turbine average hub height. We use a wind profile power law at neutral stability conditions^20^ to adjust wind speed to the wind turbine hub height (115 m in 2020 and 156 m in 2040). Wind speed in time at each site is assumed to be static in the studied period (2020–2040). We calculate the shortest distance to shore by using the distance function from the Terra package v1.5–21^21^ for R. We use the bathymetry data from ETOPO^22^ and calculate the average water depth for each pixel.

### 2.6 Modeling of Technology Mix (Technological Perspective)

We model the technology mix based on multiple OWE factors, including turbine size and lifetime, market share of technologies in the nacelle, rotor, and tower, maintenance times, replacement rates, and transportation strategies (see Table 1). The current technology mix represents an estimation for 2020.^7,8^ We use the projected values of turbine size and lifetime in 2040,^7,8^ estimates of market share of technologies in the nacelle, rotor, and tower, and maintenance times, replacement rates, and transportation strategies in 2040 based on an advanced technology scenario as the future, emergent technology mix^8^ (Table 1). We also take into account improvement of the background system, e.g., the energy mix for producing turbine components,^8^ by implementing the SSP2-RCP2.6 scenario^23^ derived by the premise framework.^24^ We refer to the summary in Table 1 and detailed data provided in previous studies.^7,8^ For simplicity, we assume that only fixed-bottom foundation technologies will be used in the North Sea. A discussion on floating foundation technologies is provided in Section 2.6 of the Supporting Information. The current existing OWFs and the planned OWFs are both modeled based on the current (2020) technology mix.

### 2.7 Sensitivity and Uncertainty Analysis

We perform a sensitivity analysis by varying the estimates of three main parameters (i.e., nominal capacity, lifetime, and wind speed) by ±20%. These parameters were varied around the future (2040) emergent technology mix, assuming the optimal siting in terms of steel and copper demand and climate change. We also perform an uncertainty analysis of the use of floating foundation technologies. Floating foundations are presumed to be applicable when the water depth exceeds 60 m. The material composition of floating foundations can be found in the source data that are provided with the paper. Moreover, we assess two end members for 2040 installed offshore wind capacity from the European Commission (215–248 GW).^25^

## 3 Results

### 3.1 OWE Locations and Environmental Footprint Based on Current Technology

**Figure 2:**
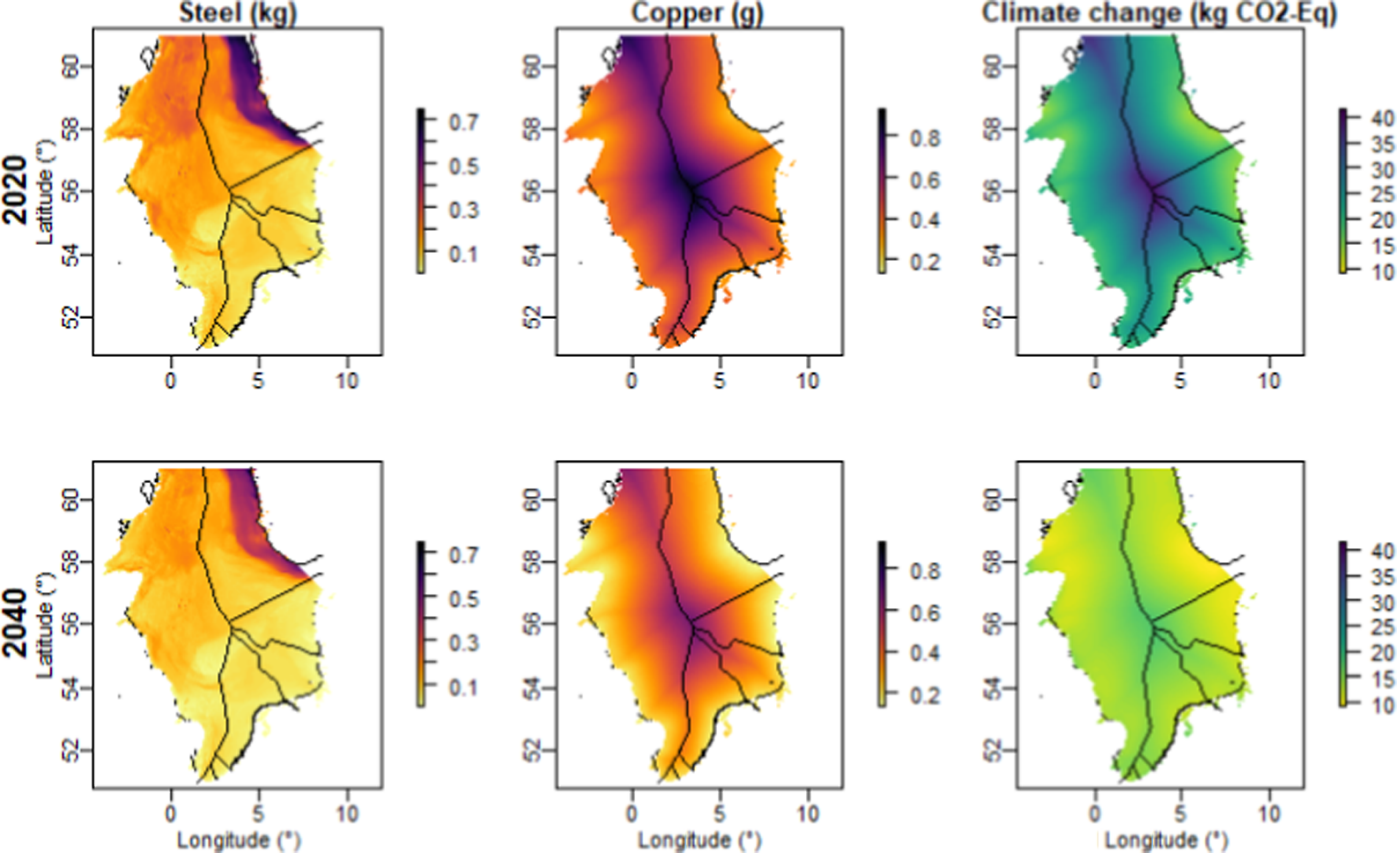
Demand for low-alloyed steel (steel) and copper per MWh of electricity production and embodied CO_2_-eq per MWh of electricity production across OWE’s full life cycle in the North Sea, based on the current (2020) and future (2040) emergent technology mix.

North Sea nearshore areas are located at shorter distances from electricity markets (Figure 1). This implies low copper and aluminum requirements for transmission and low CO_2_-eq and marine eutrophication per MWh of electricity produced compared to other regions (Figures 2, S2, and S3). OWFs located further offshore require a higher demand for copper and aluminum (Figures 2 and S2) due to increased transmission infrastructure. Copper and aluminum demands in transmission infrastructures, particularly export cables, follow a similar trend as they are both calculated based on distance from shore. OWFs further offshore also require more transport for operations and maintenance and incur additional environmental impacts related to these activities. As such, OWE placed in the central North Sea incur higher embodied CO_2_-eq and marine eutrophication per MWh of electricity produced throughout the full turbine life cycle (see Figures 2 and S3), despite the favorable wind resources and relatively shallow waters in the region (Figure S1).

The northern North Sea, especially northeastern nearshore areas (i.e., the Norwegian North Sea), exhibit fairly deep waters (Figure S1 and Table S1), which leads to a high demand for steel. Developing OWFs in the northern North Sea entails ∼4 times higher demand for steel compared to that in the southern North Sea (see Table S2 and Figures 2 and S2). However, this region benefits from favorable wind resources, resulting in a comparable demand for copper and aluminum, similar CO_2_-eq and marine eutrophication per MWh of electricity produced than other nearshore areas (Tables 2 and S2 and Figures 2, S2, and S3). Moreover, the northern North Sea regions exhibit lower marine ecotoxicity impacts compared with those in the southern North Sea (Figure S3).

**Table 2:** Average Environmental Footprints, i.e., Steel and Copper Demand and Climate Change, per MWh of Electricity Production of Existing and In-Development (In-Dev) OWFs Based on the Current (2020) OWE Technology Mix (See Table 1) and Future OWFs Based on the Future (2040) Emergent OWE Technology Mix (See Table 1) by EEZ in the North Sea^a^

exclusive economic zone (EEZ)	steel (kg)			copper (g)			climate change (kg CO_2_-eq)		
	2020		2040	2020		2040	2020		2040
	existing	in-dev	future	existing	in-dev	future	existing	in-dev	future
Belgian	0.07	0.06	0.05 (−31%)	0.45	0.42	0.26 (−40%)	22.72	21.78	14.75 (−33%)
Danish	0.03	0.07	0.05 (−30%)	0.33	0.43	0.33 (−31%)	16.73	20.71	12.60 (−44%)
Dutch	0.08	0.07	0.05 (−30%)	0.44	0.43	0.35 (−32%)	22.18	21.58	14.39 (−43%)
French		0.04	0.05 (−31%)		0.38	0.23 (−42%)		20.52	14.34 (−31%)
German	0.07	0.08	0.05 (−31%)	0.44	0.51	0.33 (−32%)	21.75	24.25	13.74 (−42%)
Norwegian		0.10	0.21 (−30%)		0.46	0.35 (−29%)		21.91	12.65 (−46%)
United Kingdom	0.07	0.12	0.11 (−29%)	0.45	0.48	0.38 (−30%)	22.35	23.11	13.87 (−45%)
the North Sea	0.06	0.08	0.11 (−30%)	0.42	0.44	0.36 (−31%)	21.15	21.98	13.52 (−41%)

a The values in brackets indicate footprint reductions compared to the associated average values in 2020.

Our results indicate existing OWFs (∼19 GW in total, see Figure 1 and Table S1) occupy locations with some of the lowest energy production to material demand or life cycle impact ratios (Table 2 and Figure S2). OWF locations in development, including those under construction, approved, and planned, show higher environmental footprints per MWh of electricity produced than existing OWF locations since they generally are located further offshore and in deeper waters (see Figure S1 and Table S1). Future OWE installations will likely move even further offshore and into deeper waters. They may thus demand more materials and result in higher CO_2_-eq per MWh of electricity produced unless higher wind speeds can lead to more efficient installations that compensate for the material demand of these locations.

**Figure 3:**
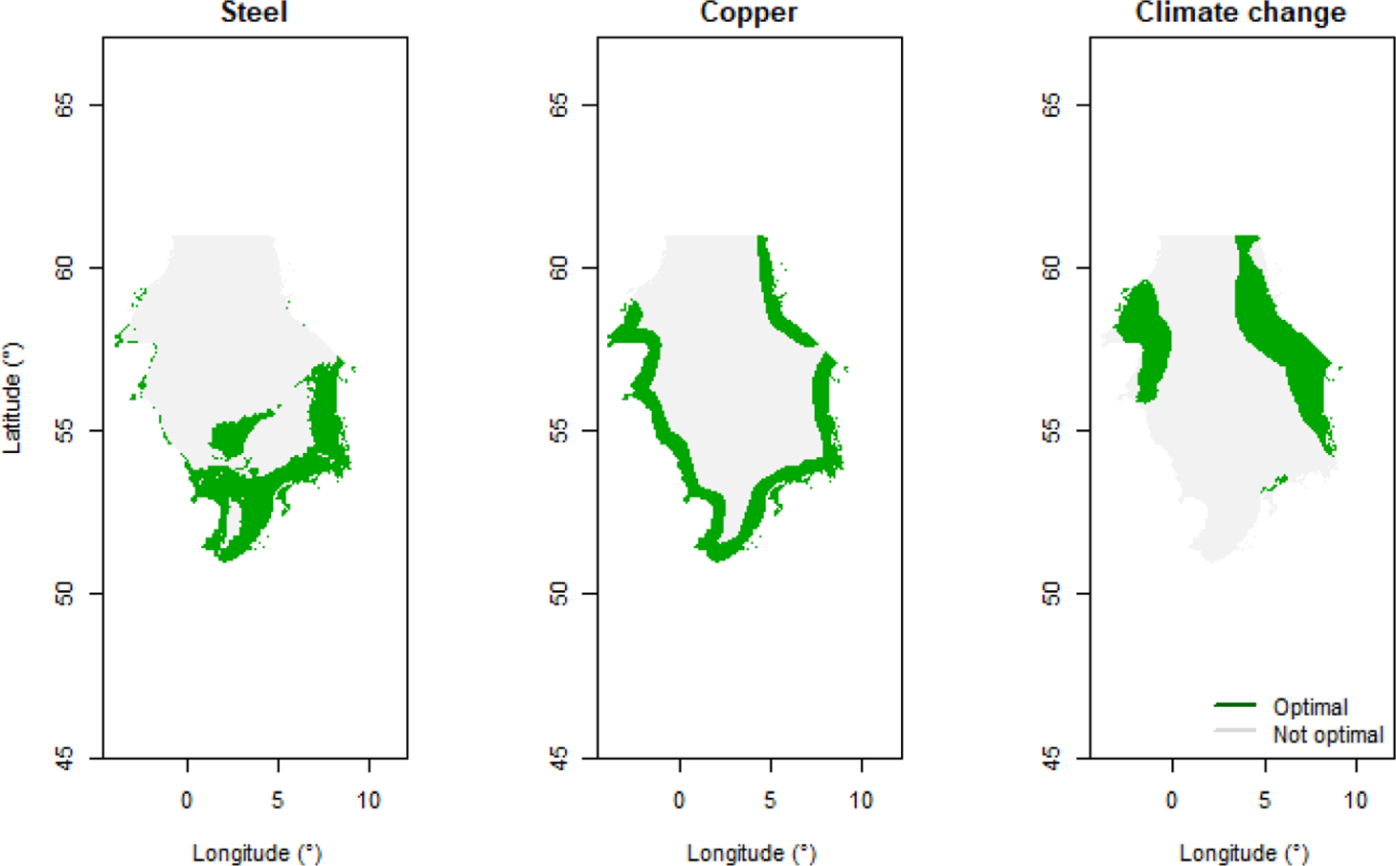
Optimal locations for installing 175 GW capacity of OWFs in terms of demand for steel and copper and climate change (embodied CO_2_-eq) per MWh of electricity produced throughout the full turbine life cycle using the future emergent technology mix.

The northern North Sea is characterized by such high wind resources (Figure S1). Our calculations show that despite higher absolute steel use, placing the OWE in this region, especially in northwestern and northeastern nearshore areas, leads to the lowest CO_2_-eq per MWh of electricity produced (Figure 3). However, these trade-offs can differ for other impact factors. Eastern nearshore, southern, and central (mainly the Dogger Bank) regions of the North Sea are the optimal locations for OWE development in terms of steel requirement (Figure 3). Locating future OWFs in nearshore regions along the North Sea coastlines will minimize copper use (Figure 3). Overall, placing OWFs in optimal locations could decrease the extent of the environmental footprint of the OWE by ∼26% to ∼65% (Table 3). The optimal locations that minimize CO_2_-eq, i.e., the eastern and northwestern nearshore areas of the North Sea (see Figure 3), could lead to ∼6.44 kg (∼26% drop) of CO_2_-eq per MWh of electricity production.

**Table 3:** Average Current (2020) and Future (2040) Environmental Footprints (i.e., Steel and Copper Demand and CO_2_-Eq.) per MWh of Electricity Production across the Full Turbine Life Cycle in the North Sea, Based on Average Values without Optimal Siting (i.e., Average) and the Optimal Siting in Terms of Steel and Copper Demand and Climate Change (Figure 3)^a^

	steel (kg)		copper (g)		climate change (kg CO_2_-eq)	
	2020	2040	2020	2040	2020	2040
average	0.17	0.11	0.51	0.36	24.49	13.52
optimal siting for steel	0.06	**0.05**	0.46	0.30	22.53	13.50
optimal siting for copper	0.18	0.15	0.35	**0.21**	18.06	12.01
optimal siting for climate change	0.24	0.20	0.37	0.24	18.05	**11.26**

a The values in bold indicate the future (2040) steel and copper demand and CO_2_-Eq. per MWh of electricity production across the full turbine life cycle in the North Sea, based on the optimal siting in terms of steel and copper demand and climate change, respectively.

### 3.2 Improvement Potential Using an Improved Technology Mix

A future emergent technology mix, which includes turbine size enlargement, lifetime extension, and component technological innovation, together with improved background energy systems toward 2040, such as the greener energy (see Table 1), has the potential to lead to the following reductions: steel demand to ∼0.06 kg (∼30% drop), copper demand to ∼0.15 g (∼31% drop), and CO_2_-eq to ∼10.97 kg (∼41% drop) per MWh of electricity production across OWE’s full life cycle (Tables 2, 3, and S3). Other environmental impacts undergo a reduction with the future emergent technology mix as well, including marine ecotoxicity and marine eutrophication (Figure S3 and Table S3). Further, developing future OWFs in the optimal locations for, respectively, steel use, copper use, and CO_2_-eq (Figure 3 and Table 3) with this future technology would lead to the following reductions: steel demand to ∼0.12 kg (∼72% drop), copper demand to ∼0.31 g (∼60% drop), and climate change to ∼13.23 kg CO_2_-eq (∼54% drop) per MWh of electricity production across OWE’s full life cycle.

### 3.3 Achieving Low-Impact OWE in Individual Exclusive Economic Zones (EEZs)

The Norwegian and Danish North Seas, among others, exhibit the lowest future impacts per MWh of electricity production (Tables 2, S2, and S3). The Norwegian and UK North Seas exhibit high steel demand and moderate CO_2_-eq impacts but low impacts on marine ecotoxicity per MWh of electricity output across the full turbine life cycle (Figures 2, S2, and S3 and Table S3). These regions currently lack operational OWFs but have massive potential for OWE development due to favorable wind resources (Figure S1 and Table S1). The Norwegian government has set an ambition to develop 4.5 GW OWFs with a specific focus on floating wind farms.^26^ As the largest OWE market in Europe, the UK has a more ambitious mission to achieve up to 50 GW of OWE by 2030.^27^ There are no planned OWFs in the Belgian and French North Sea areas due to limited space (Figure 1). The Belgian, Danish, Dutch, and German EEZs partially overlap with protected biodiversity areas, indicating high levels of potential biodiversity impacts (Figure 1 and Table S3). The Dutch and German EEZs exhibit moderate levels of material demand and life cycle CO_2_-eq impacts per MWh of electricity production.

### 3.4 Cumulative Footprints

Deploying 175 GW of the OWE capacity in the North Sea, capable of generating ∼12,223 TWh of electricity by 2040 with current technologies, will require ∼2.1 Mt of steel and ∼6.2 kt of copper. In this calculation, we use the average wind speed, water depth, and distance from shore of the North Sea for each grid in the GIS map. The cumulative embodied CO_2_-eq for this OWE capacity is ∼299 Mt of CO_2_-eq throughout the full 20 year turbine life cycle (24.49 kg CO_2_-eq/MWh). These limited CO_2_-eq compare favorably with the 4767 Mt CO_2_-eq (∼16 times more) (390 kg of CO_2_-eq/MWh) that are currently produced annually for generating the same amount of electricity with the 2020 continental European electricity mix.

The technological development could lead to a ∼1.5 Mt and ∼4.3 kt demand for steel and copper and ∼176 Mt CO_2_-eq for installing 175 GW OWFs, based on the average wind speed, water depth, and distance from shore of the North Sea. Considering that 175 GW OWFs will be installed between 2020 and 2040, on average, the annual demand for steel and copper accounts for ∼0.6% and ∼0.7% of the global steel and copper consumption in 2020,^28,29^ respectively. Optimizing the OWE placement for minimal steel and copper requirements (see Table 3) leads to only ∼0.7 Mt of steel and ∼2.5 kt of copper demand throughout the 25 year full life cycle. These locations would incur a cumulative CO_2_-eq of 156–175 Mt (Table 3). Optimizing wind turbine siting based on minimal CO_2_-eq leads to only 146 Mt of cumulative CO_2_-eq.

### 3.5 Sensitivity and Uncertainty Analysis Results

The largest variations of footprints are related to wind resources. Climate change impacts have an increase of ∼68% when the wind speed decreases by 20% (Table S4). In this event, steel and copper requirements undergo an increase of approximately 89% and 61%, respectively. Conversely, a 20% increase in wind speed could reduce the demand for steel and copper and climate change by ∼44%, ∼47%, and ∼39%, respectively (Table S4). Turbine size has a more attenuated effect since a larger nominal capacity leads to a higher harvesting of wind resources per MW. The climate change impacts will decrease by ∼37% if the proposed nominal capacity increases by 20% (and increase by ∼39% when the proposed nominal capacity decreases by 20%). The lifetime variations can have a substantial effect on the environmental footprints. Steel demand, copper demand, and CO_2_-eq change by approximately +28% (–17%), +27% (–18%), and +38% (–20%) when lifetime decreases (or increases) by 20%, respectively.

Floating OWE technologies present an opportunity to harness wind energy in deep waters, having the potential to halve the steel requirement relative to fixed-bottom foundations in northern locations (refer to Figure S4). An increase in future OWE capacity from 175 GW to 215–248 GW leads to a larger occupation of the North Sea (see Figures S5 and S6). The optimal maps for steel and copper demand, as well as climate change impact (measured in embodied CO_2_-eq) per MWh of electricity produced across the full turbine life cycle using future technology, exhibit patterns similar to those observed in the 175 GW installation scenario (Figures S5 and S6).

## 4 Discussion

### 4.1 OWE-Related Biodiversity Impacts

Potential OWE developments in the central North Sea are located near protected areas (Figure 1). In particular, OWE development near and in the Dogger Bank in the central North Sea needs to carefully address ecological concerns.^30^ Future installations in central areas of the North Sea should focus on efficient operation and maintenance practices (e.g., detailed vessel routing planning) and component technological advancements (e.g., more usage of direct drive nacelles with fewer failure rates^31^). Such improvements, however, also reduce embodied impacts of the OWE on other locations, implying that the relative drawbacks of the development of the OWE in the Dogger Bank will persist. Floating wind technologies^32^ present an opportunity to harness wind energy in deep waters, having the potential to halve the steel requirement relative to fixed-bottom foundations in northern locations (Figure S4). Moreover, floating OWFs exert fewer impacts on marine biodiversity due to reduced steel usage for foundation infrastructure. Although the construction of anchors might require pile driving, the smaller size of these anchors results in comparatively limited affected areas and associated biodiversity impacts compared to fixed-bottom foundations.^33^

Larger turbines lead to biodiversity benefits. Although they require increased spacing and thus occupy larger seabed areas per turbine unit, the impacts per MW on marine biodiversity will be substantially lower.^34^ Further, the tip speed is restricted to approximately 90 m/s to mitigate blade erosion. Consequently, wind turbines with longer blades operate at lower rotational speeds, which reduces the incidence of collisions with birds and bats.^35^ Turbine lifetime extensions by 20% could cut down CO_2_-eq per MWh of electricity produced by ∼20% (Table S4). Moreover, circular designs such as closed-loop recycling^8^ can supply secondary materials, mitigate material criticality, and lead to a further reduction of 6–9% CO_2_-eq per MWh of electricity produced.

### 4.2 Limitations and Further Research

We modeled electricity output from wind turbines in a simplified approach by using the Rayleigh statistics of average annual wind speeds at the site. Future research could enhance the accuracy of electricity output estimates by incorporating more precise assessments of wind speed variations and distribution patterns. We evaluated only one specific change in future technologies. However, larger turbine sizes (currently limited by engineering constraints) may be developed.^36^ In addition, low-maturity technological component advancements, such as floating foundations, could develop faster than expected.^37^ Future research should be conducted to deepen the understanding of floating foundation design, mooring systems, and dynamic cables, especially as more OWFs move further offshore into deeper waters. Several components of wind turbines can be made with lighter designs and materials in an attempt to reduce environmental impacts while achieving structural fatigue requirements and maintaining strength.^38^ Furthermore, a faster decarbonization of the background energy system would lead to a further lowering of embodied CO_2_-eq of OWFs. To further mitigate footprints that are not reliant on the optimal spatial siting of OWFs, it is crucial to make substantial investments in technological advancement of the OWE, such as turbine size enlargement, lifetime extension, and component technological innovations. More in-depth follow-up research that looks better at such technical development choices via multicriteria analysis is desired.

Biodiversity impacts of the OWE were assessed only via potential overlaps with protected areas. However, OWE operation could also lead to positive impacts.^34^ More research is required to better understand the ecological mechanism of the biodiversity change in OWFs. OWE-driven biodiversity changes should be contextualized around other societal and industrial activities (e.g., oil and gas, fisheries, and tourism). More monitoring efforts have to be done to further understand the co-use of OWFs with other activities since these aspects of potential environmental impacts associated with OWE development are still largely unexplored in current OWE planning.^39^ Moreover, our work adds more value to the OWE planning, providing a steppingstone toward a better understanding of the broad range of environmental impacts. This could be achieved through marine spatial planning processes that consider ecosystem services.^40^

### 4.3 Policy Implications

A sound spatial planning of the 175 GW OWFs envisaged by 2040 in the North Sea is crucial. Different siting locations have trade-offs in terms of material use and life cycle environmental impacts. Comprehensive studies in which such spatial trade-offs are optimized are lacking. Assessing these trade-offs and complementing them with macro-level collaborations across countries responsible for the different EEZs can lead to optimal siting decisions in infrastructure development that ultimately will cover 25% of the North Sea surface.

Overall, there is a lack of macro-level collaboration among countries concerning spatial planning for OWE in the North Sea.^41^ To achieve minimal impacts, cross-border spatial planning and collaboration between various countries are crucial, which requires aligning various policies and targets. For example, the Strategic Environmental Assessment North Sea Energy^42^ was carried out to assess the cumulative impacts of large-scale wind farms, involving cross-border maritime spatial planning among authorities in the Netherlands, Germany, France, Scotland, and Denmark. The Ostend declaration by energy ministers^43^ outlines a commitment to transform the North Sea into Europe’s green power hub through cross-border renewable energy projects. The knowledge community “North Sea Shipping Group” (NSSG) was created to exchange experiences and knowledge about OWFs in the North Sea.^44^ Further, it is important to examine the conflicts with marine activities, such as navigation routes, underwater pipelines, fisheries, sand mining areas, military training zones, tourism, and the preservation of underwater cultural heritage, including archeological investigations. As future OWFs are likely to occupy one-fourth of the seabed space, the possibilities of multifunctional use of these areas should be carefully considered. This requires new regulations and technical innovations allowing for the multifunctional use of areas in the North Sea. Such innovations include the construction of oyster beds,^45^ floating solar farms,^46^ mussel farms or seaweed cultivation^47^ between wind turbines’ supporting structures, coexistence with fishing activities,^48^ and artificial energy islands.^49^

## Data Availability

Source data is provided with the paper.

## Supporting Information Available

Source data (XLSX)
More detailed documentation of methods; geographic characteristics of the North Sea; demand for low-alloyed steel, copper, and aluminum; life cycle environmental impacts; demand for steel per MWh of electricity production across the full turbine life cycle in the North Sea; optimal locations for installing 215 and 248 GW capacities of OWFs; geographic characteristics of OWE development; and data on environmental footprints (PDF)
