# Supplementary material for: Optimal Environmental Siting of Future Wind Turbines in the North Sea
Source: Environ Sci Technol. 2024 Dec 19;58(52):22944–52. doi: 10.1021/acs.est.4c03861 (PMC11697346; doi:10.1021/acs.est.4c03861)
Supplement: Supplementary file 2 — es4c03861_si_002.pdf [file es4c03861_si_002.pdf]

Supplementary materials

Optimal environmental siting of future wind turbines in the North Sea

Chen Li[1]*, Bernhard Steubing[1], Joeri Morpurgo[1], Arnold Tukker[1,2], José M. Mogollón[1]

[1] Institute of Environmental Sciences (CML), Leiden University, P.O. Box 9518, 2300 RA Leiden, the Netherlands

[2] Netherlands Organization for Applied Scientific Research, P.O. Box 96800, 2509 JE Den Haag, the Netherlands

* Corresponding author: c.li@cml.leidenuniv.nl

Summary: 8 pages, 6 figures, and 4 tables.

2. Materials and methods

2.1 Calculation of electricity output

The electricity output is calculated based on the production curve and a probability density function that reflects the site-specific average wind speed [1]. For simplicity, we estimate the electricity production of a single wind turbine across its lifetime by using its nominal capacity and lifetime, and a Rayleigh statistics. The Rayleigh statistics is determined by a Rayleigh distribution, which models the wind speed distribution at hub height, along with the turbine's power curve [1].

2.2 Calculation of material demand

We consider three materials, i.e. steel, copper and aluminum, in this study. There are two types of foundations, i.e. fixed-bottom based and floating foundations. The floating foundations are suitable for deep waters, but the North Sea is characterized by relatively shallow waters. Further, floating foundation technologies are still evolving and have not been deployed at a commercial scale. Monopile foundations dominate the global fixed-bottom based foundation market (> 80%) and were widely used in the North Sea. Therefore, Monopile foundations are assumed to be used in the North Sea. Material requirement for Monopile foundations is based on foundation weight (FW, in tons) and Fe intensity (100% of low alloyed steel). FW is found linear to foundation diameter (FD, in meters) and water depth (WD, in meters).

$$\boldsymbol{FW} = 137.92 + 2.27 \times \left(\frac{\boldsymbol{FD}}{\mathbf{2}}\right)^2 \times \boldsymbol{WD} \tag{1}$$

This estimation is from the statistical analysis of foundation parameters (see Source data) based on multiple historical offshore wind farms [2].

Material requirement for inter array cables is modelled in line with [3]. Demand for copper, and aluminium from export transmission cables is calculated based on distance from shore and associated material intensity [3].

2.3 Calculation of life cycle impacts on the climate

We consider climate change, marine ecotoxicity and marine eutrophication as representatives of key environmental impact categories. OWE is key to energy transition and considered as a promising renewable energy source to mitigate greenhouse gases emissions (GHGs). Climate change is a widely used impact category to represent GHGs. OWE is located over shallow open waters in the sea and moving further into deep waters. Marine ecotoxicity and marine eutrophication are two impact categories directly linked to marine environment. Although the risks of marine ecotoxicity and marine eutrophication are considered low in wind turbine manufacturing, especially compared to the pollution from fossil fuel extraction and use, the installation and maintenance of offshore wind turbines involve the use of materials like metals, lubricants, and anti-corrosion coatings. These impacts must therefore be carefully considered and regulated.

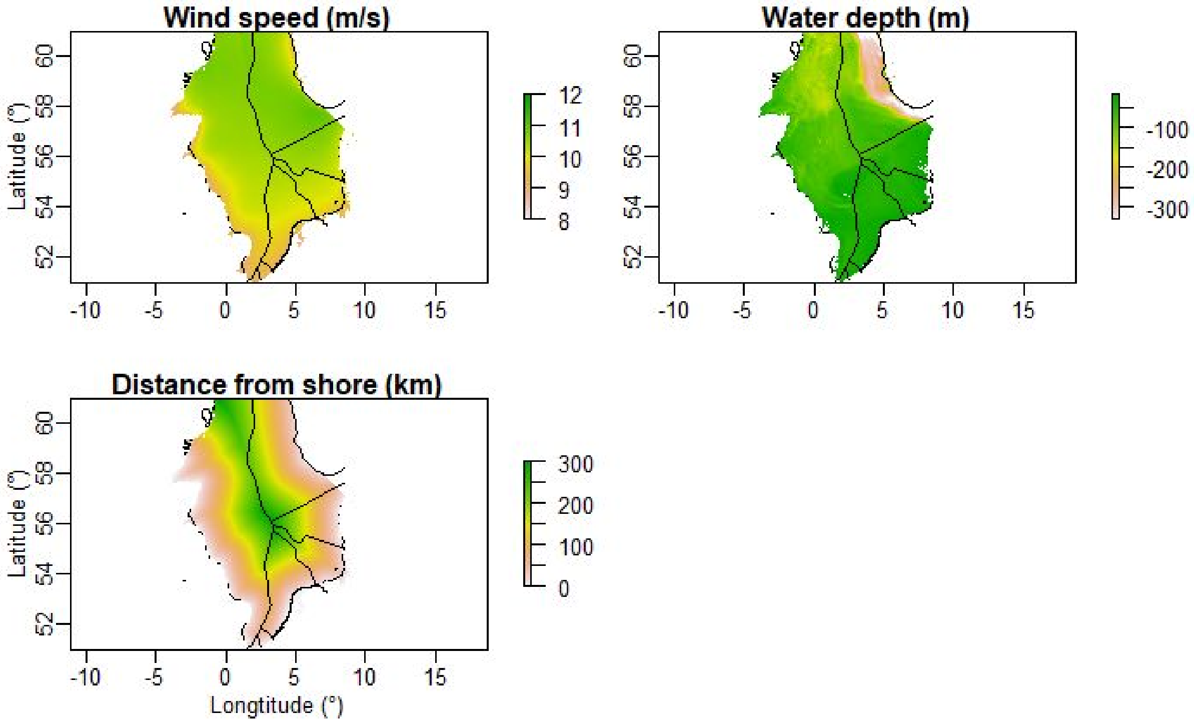

Figure S1: Geographic characteristics of the North Sea, including wind speed, water depth, and distance from shore.

2. Results

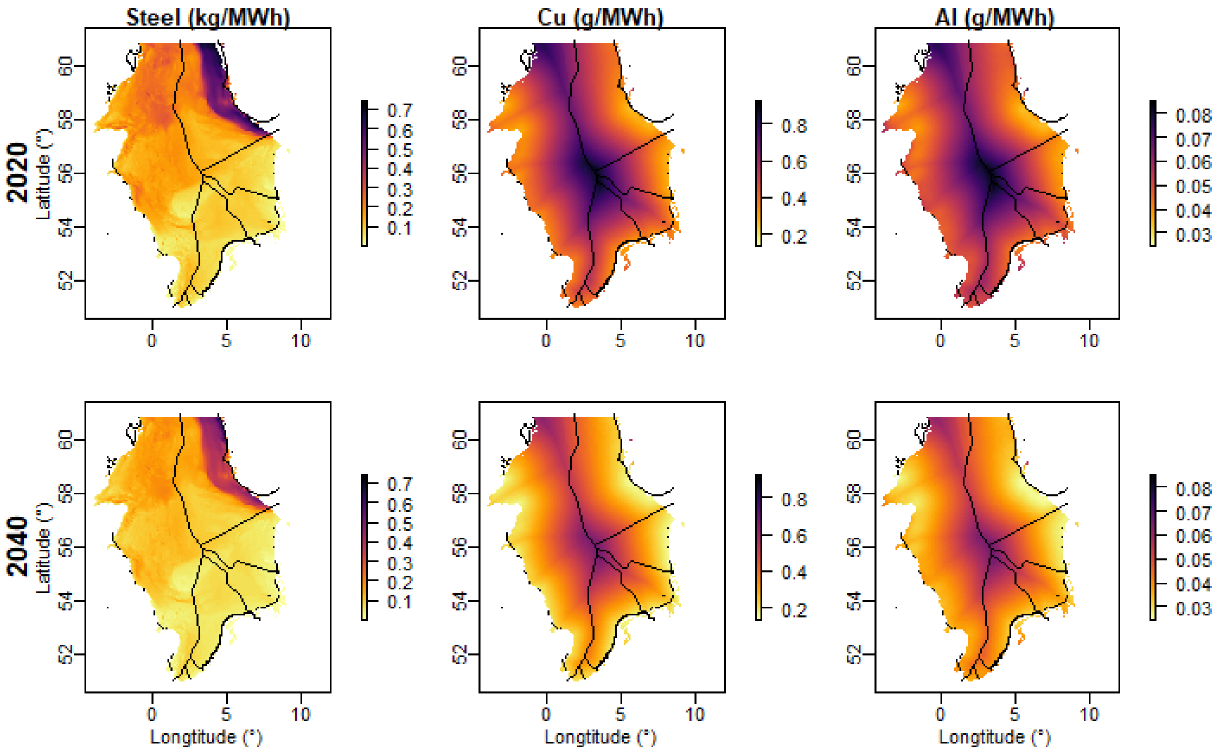

Figure S2: Demand for low-alloyed steel (steel), copper (Cu), and aluminum (Al) per MWh electricity production across OWE full life cycle in the North Sea, based on current (2020) and future (2040) technology mix.

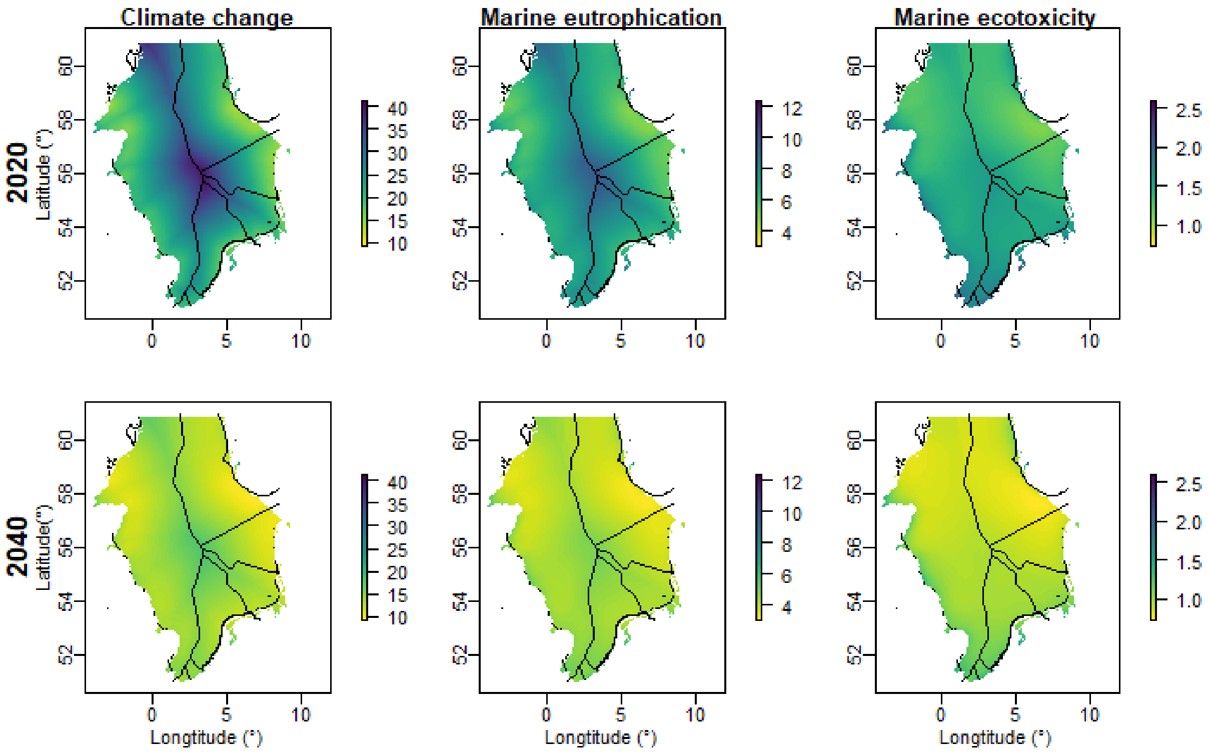

Figure S3: Life cycle environmental impacts, i.e. climate change (kg CO_2-Eq/MWh), marine ecotoxicity (kg 1,4-DC. /MWh), and marine eutrophication (g N-Eq 46/MWh), based on current (2020) and future (2040) technology mix.

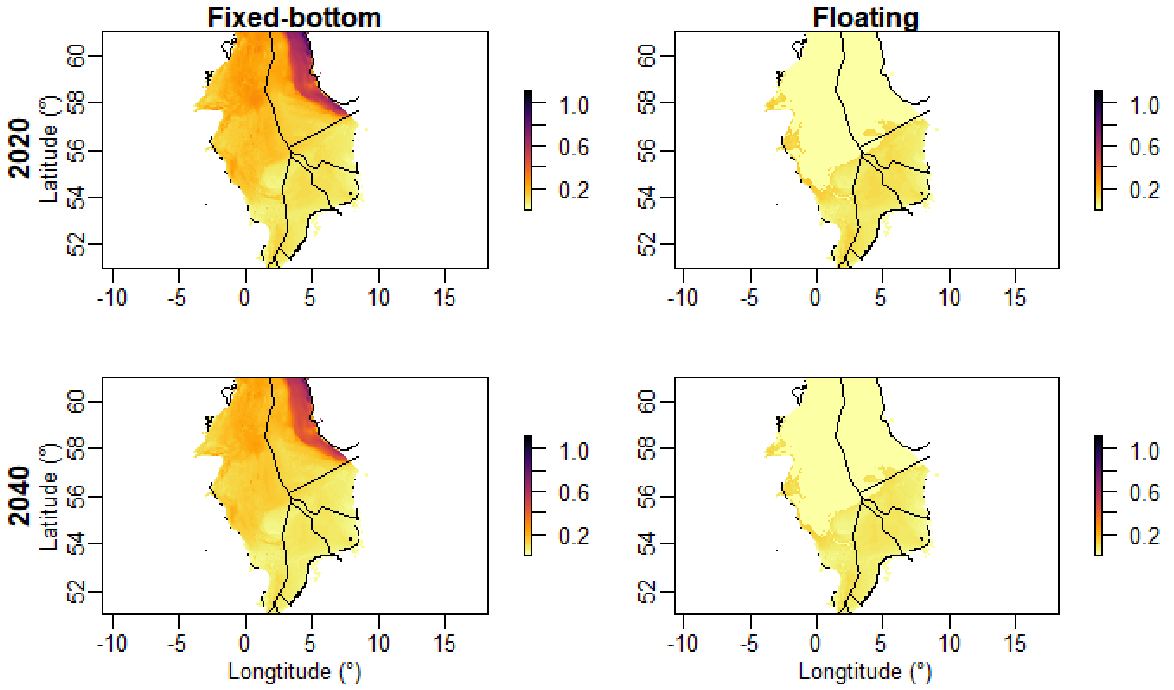

Figure S4: Demand for steel per MWh electricity production across full turbine life cycle in the North Sea, based on floating foundation technology development from current (2020) to the future (2040).

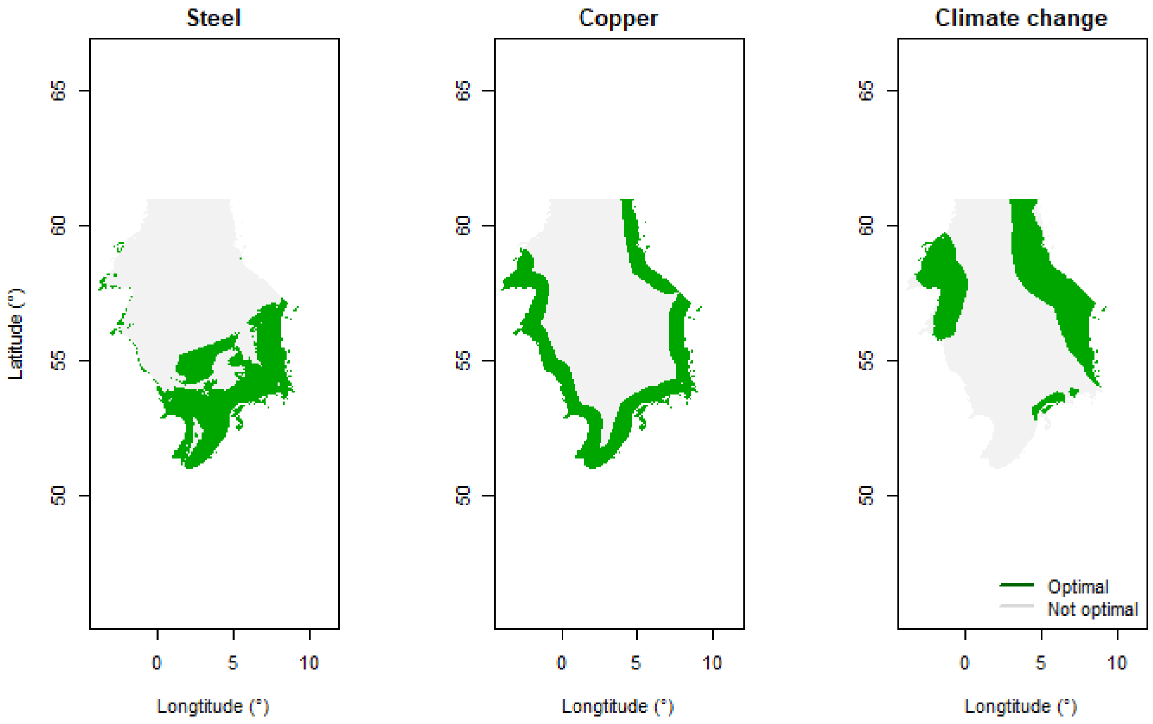

Figure S5: The optimal locations for installing 215 GW capacity of OWFs in terms of demand for steel and copper, and climate change (embodied CO_2-eq.) per MWh electricity produced throughout the full turbine life cycle using future technology mix.

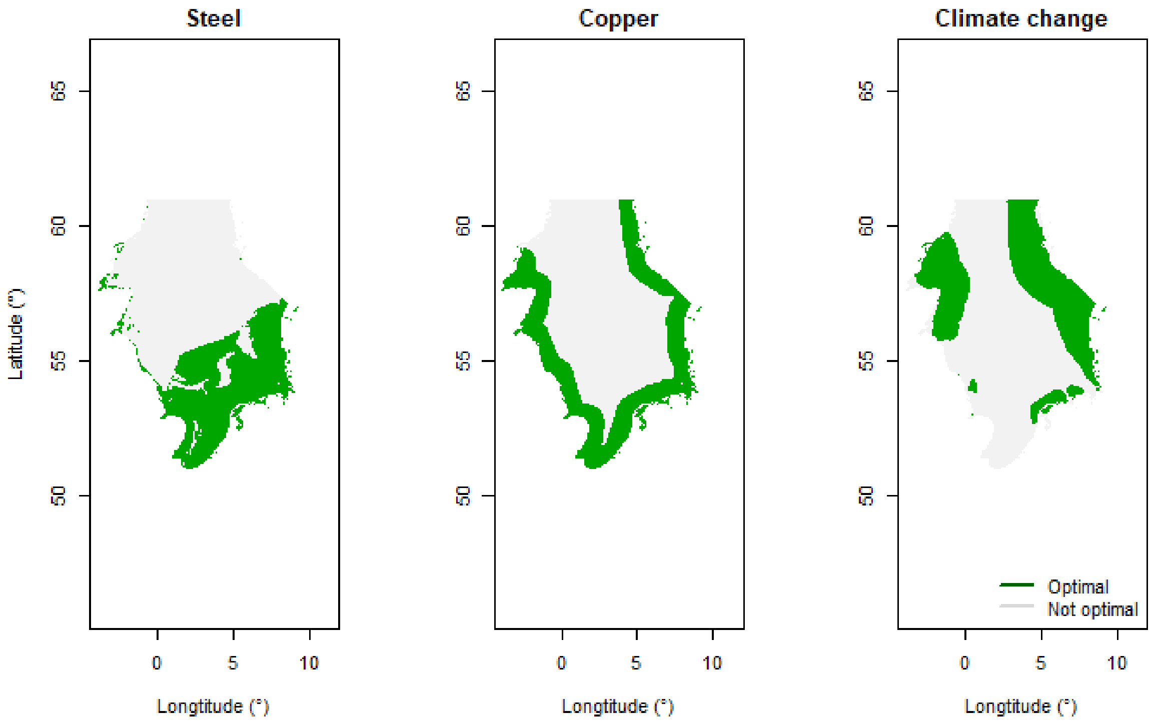

Figure S6: The optimal locations for installing 248 GW capacity of OWFs in terms of demand for steel and copper, and climate change (embodied CO_2-eq.) per MWh electricity produced throughout the full turbine life cycle using future technology mix.

Table S1: Geographic characteristics of OWE development, and existing and in-development (In-dev) offshore wind farms by EEZ in the North Sea.

	**Belgian**		**Danish**		**Dutch**		**French**		**German**		**Norwegian**		**United Kingdom**	
Wind speed (m/s)	9.3		10.5		10.0		9.3		10.1		10.7		10.3	
Distance from shore (km)	29.5		107.5		97.3		16.7		91.1		128.7		129.5	
Water depth (m)	-23.5		-39.5		-32.3		-24.8		-30.0		-155.7		-77.7	
	Existing	In-dev	Existing	In-dev	Existing	In-dev	Existing	In-dev	Existing	In-dev	Existing	In-dev	Existing	In-dev
Wind speed (m/s)	9.4		10.3		9.5		-		10.0		-		9.9	
Distance from shore (km)	38.8	29.5	29.1	90.9	41.1	46.4	-	5.8	67.4	95.3	-	123.2	65.1	100.3
Water depth (m)	-28.0	-22.9	-13.2	-38.5	-29.0	-28.1	-	-14.6	-30.8	-38.5	-	-54.1	-33.1	-61.4

Table S2: The average environmental footprints (material demand and life cycle impacts) per MWh electricity production of existing and in-development (In-dev), including under construction, approved, and planned OWFs (see Figure 1) by EEZ in the North Sea, based on the current (2020) OWE technology mix.

**Exclusive economic zone (EEZ)**	**Material demand**						**Life cycle impact categories**					
	Steel (kg)		Copper (g)		Aluminium (g)		Climate change (kg CO_2-Eq)		Marine ecotoxicology (kg 1,4-DC.)		Marine eutrophication (g N-Eq)	
	Existing	In-dev	Existing	In-dev	Existing	In-dev	Existing	In-dev	Existing	In-dev	Existing	In-dev
**Belgian**	0.07	0.06	0.45	0.42	0.05	0.05	22.72	21.78	1.73	1.80	7.48	7.48
**Danish**	0.03	0.07	0.33	0.43	0.04	0.05	16.73	20.71	1.32	1.29	5.62	6.20
**Dutch**	0.08	0.07	0.44	0.43	0.05	0.05	22.18	21.58	1.67	1.59	7.25	7.00
**French**		0.04		0.38		0.05		20.52		1.84		7.36
**German**	0.07	0.08	0.44	0.51	0.05	0.06	21.75	24.25	1.47	1.49	6.75	7.19
**Norwegian**		0.10		0.46		0.05		21.91		1.24		6.27
**United Kingdom**	0.07	0.12	0.45	0.48	0.05	0.05	22.35	23.11	1.53	1.41	6.98	6.83
**The North Sea**	0.06	0.08	0.42	0.44	0.05	0.05	21.15	21.98	1.54	1.52	6.82	6.90

Table S3: The average environmental footprints (material demand and life cycle impacts) per MWh electricity production of developing OWFs in each EEZ in the North Sea, based on current (2020) and future (2040) OWE technology mix. [1] High: more overlaps with protected areas; Low: less overlaps with protected areas.

Exclusive economic zone (EEZ)	Material demand						Life cycle impact categories						Potential biodiversity loss[1]
	Steel (kg)		Copper (g)		Aluminium (g)		Climate change (kg CO_2-Eq)		Marine ecotoxicology (kg 1,4-DC.)		Marine eutrophication (g N-Eq)		
	2020	2040	2020	2040	2020	2040	2020	2040	2020	2040	2020	2040	
**Belgian**	0.07	0.05	0.43	0.26	0.05	0.04	22.18	14.75	1.76	1.21	7.46	4.90	High
**Danish**	0.08	0.05	0.47	0.33	0.05	0.04	22.52	12.60	1.34	0.88	6.59	3.88	High
**Dutch**	0.07	0.05	0.52	0.35	0.06	0.04	25.09	14.39	1.56	1.03	7.49	4.48	High
**French**	0,07	0.05	0.40	0.23	0.05	0.04	20.75	14.34	1.75	1.21	7.20	4.83	Low
**German**	0,07	0.05	0.49	0.33	0.05	0.04	23.78	13.74	1.50	0.99	7.14	4.30	High
**Norwegian**	0,30	0.21	0.49	0.35	0.05	0.04	23.38	12.65	1.31	0.85	6.66	3.83	Low
**United Kingdom**	0,15	0.11	0.54	0.38	0.06	0.04	25.43	13.87	1.44	0.94	7.29	4.21	Low
**The North Sea**	0.17	0.11	0.51	0.36	0.05	0.04	24.49	13.52	1.47	1.01	6.98	4.28	-

Table S4: Future (2040) environmental footprints (i.e. steel and copper demand, and CO_2-Eq.) variations per MWh electricity production across full turbine life cycle in the North Sea considering parameter changes, based on the optimal siting in terms of steel and copper demand, and climate change, respectively.

	**Steel (kg), optimal siting for steel**		**Copper (g), optimal siting for copper**		**Climate change (kg CO_2-Eq.), optimal siting for climate change**	
	Value	Variation	Value	Variation	Value	Variation
Base case	0.05		0.21		11.26	
Nominal capacity, -20%	0.09	+75%	0.32	+51%	15.65	+39%
Nominal capacity, +20%	0.03	-35%	0.15	-28%	7.09	-37%
Lifetime, -20%	0.08	+28%	0.27	+27%	15.54	+38%
Lifetime, +20%	0.04	-17%	0.17	-18%	9.08	-20%
Wind speed, -20%	0.10	+89%	0.34	+61%	18.92	+68%
Wind speed, +20%	0.03	-44%	0.11	-47%	6.87	-39%

References

1. Hansen, M. O. L. (2008). *Aerodynamics of Wind Turbines: second edition.* (2 ed.) Earthscan.
2. Negro, V., et al. (2017). Monopiles in offshore wind: Preliminary estimate of main dimensions. Ocean Engineering, 133, 253-261.
3. Chen, Z.Y., Kleijn, R., & Lin, H.X. (2022). Metal Requirements for Building Electrical Grid Systems of Global Wind Power and Utility-Scale Solar Photovoltaic until 2050. Environmental Science & Technology.
4. Li, C., et al. (2022). Future material requirements for global sustainable offshore wind energy development. Renewable & Sustainable Energy Reviews, 164.
5. Li, C., et al. (2022). Environmental Impacts of Global Offshore Wind Energy Development until 2040. Environmental Science & Technology, 56(16), 11567-11577.
